# Res@LDH: A Novel Nanohybrid Therapeutic for Ischemia–Reperfusion Injury with Dual Reactive Oxygen Species Scavenging Efficiency

**DOI:** 10.34133/bmr.0108

**Published:** 2024-12-03

**Authors:** Min Liu, Siyuan Liu, Yafan Bai, Mingru Zhang, Duo Zhang, Ruijin Sun, Guyan Wang, Yulong Ma

**Affiliations:** ^1^ Department of Anesthesiology, Beijing Tongren Hospital, Capital Medical University, Beijing 100730, China.; ^2^ Department of Anesthesiology, Affiliated Hospital of Nantong University, Nantong 226001, China.; ^3^ School of Science, China University of Geosciences, Beijing (CUGB), Beijing 100083, China.; ^4^ Department of Anesthesiology, The First Medical Center of Chinese PLA General Hospital, Beijing 100853, China.

## Abstract

Ischemic stroke poses a global health challenge, necessitating effective therapeutic interventions given the limited time window for thrombolytic therapy. Here, we present Res@LDH, a novel nanohybrid therapeutic agent boasting a dual reactive oxygen species scavenging efficiency of approximately 90%. Comprising Ge-containing layered double hydroxide nanosheets (Ge-LDH) as a drug nanocarrier and resveratrol as a neuroprotective agent, Res@LDH demonstrates enhanced permeability across the blood–brain barrier, ensuring high biocompatibility and stability. We explored the potential of Res@LDH in mitigating oxidative stress injury induced by middle cerebral artery occlusion and reperfusion in mice, as well as H_2_O_2_-induced cytotoxicity in HT22 cells. Our experiments unveil Res@LDH’s capacity to ameliorate neurological deficits, reduce the infarction volume, mitigate blood–brain barrier disruption, exhibit a robust antioxidant activity, and dampen the release of proinflammatory cytokines. Moreover, Res@LDH treatment markedly attenuates microglial and astrocytic activation. Leveraging a pioneering synthetic approach harnessing Ge-LDH and resveratrol, Res@LDH emerges as a promising strategy for addressing ischemia–reperfusion injury, offering a concise solution to current therapeutic challenges.

## Introduction

Ischemic stroke, which constitutes approximately 80% of all stroke cases globally, stands as a leading cause of mortality and disability worldwide, with over 10 million new cases annually [1–4]. Effective therapeutic strategies such as thrombolysis and thrombectomy aimed at achieving early vascular recanalization have been validated [5–8]. Intravenous recombinant tissue plasminogen activator administration alone or in combination with various mechanical thrombectomy devices is approved by the US Food and Drug Administration and represents a cornerstone of treatment for ischemic stroke [6]. However, the narrow time window of 4.5 to 6 h for effective thrombolytic therapy restricts its accessibility to only a minority of patients [5,9,10]. Therefore, there is a critical need to explore novel therapies for ischemic stroke.

Numerous studies underscore oxidative stress as a principal contributor to ischemic stroke injury [11–13]. Regardless of whether a patient undergoes thrombolytic therapy for restoring cerebral blood flow (CBF), they remain susceptible to both ischemic stroke and reperfusion injury [14]. This susceptibility arises from the overproduction of reactive oxygen species (ROS), including hydroxyl radicals (•OH), superoxide anions (O_2_^•−^), hydrogen peroxide (H_2_O_2_), and nitric oxide (NO), within the ischemic penumbra [15–17]. Moreover, excessive ROS generation triggers a cascade of cellular signaling pathways, exacerbating blood–brain barrier (BBB) permeability damage, brain edema, and inflammation and ultimately culminating in neuronal cell death and brain tissue damage [17–20]. Consequently, scavenging ROS emerges as a critical therapeutic approach for ischemic stroke injury.

Nanotechnology presents a promising frontier in ischemic stroke treatment owing to its capability to combat ROS and its miniature size. Nanomaterials such as cerium dioxide (CeO_2_), manganese, carbon, and copper oxide (CuO) nanoparticles have shown potential in this regard [21–24]. Notably, layered double hydroxides (LDHs) also possess a small size and other excellent properties, including a harmonized structure, biocompatibility, scalable production, and economical preparation [25–28]. LDHs have been demonstrated to possess antioxidant activity by incorporation of appropriate metal ions into their structure, such as copper, manganese, ruthenium, gadolinium, and iron [25–31]. Previous studies have reported that copper-incorporated LDHs mitigate oxidative stress [26], and atorvastatin–ferritin gadolinium LDHs have been shown to play a crucial role in ischemic stroke by oxidative stress inhibition [25]. Despite marked strides in the field, LDH-based nanomaterials still present challenges concerning structural and functional stability. Given the potential of incorporating different metal ions into LDH nanoparticles, we investigated the role of germanium (Ge)-doped LDH in ischemic stroke. Herein, Ge-doped LDH nanoparticles emerge as promising candidates for mitigating ROS in ischemic stroke injury.

Resveratrol (Res), a natural polyphenolic compound abundant in grapes and red wine, exerts crucial neuroprotective effects in ischemic stroke by inhibiting oxidative stress and inflammation [32–34]. However, its application is greatly hampered by its low water solubility, poor bioavailability, and challenges in traversing the BBB to reach ischemic brain tissue [35,36]. Addressing these challenges, the use of Ge-doped LDH nanomaterials may offer a solution to enhance the bioavailability and efficacy of Res while potentially reducing costs in ischemic stroke treatment. Despite this potential, studies employing Ge-doped LDH nanomaterials as drug carriers for Res in ischemic stroke treatment remain scarce.

**Fig. 1.**
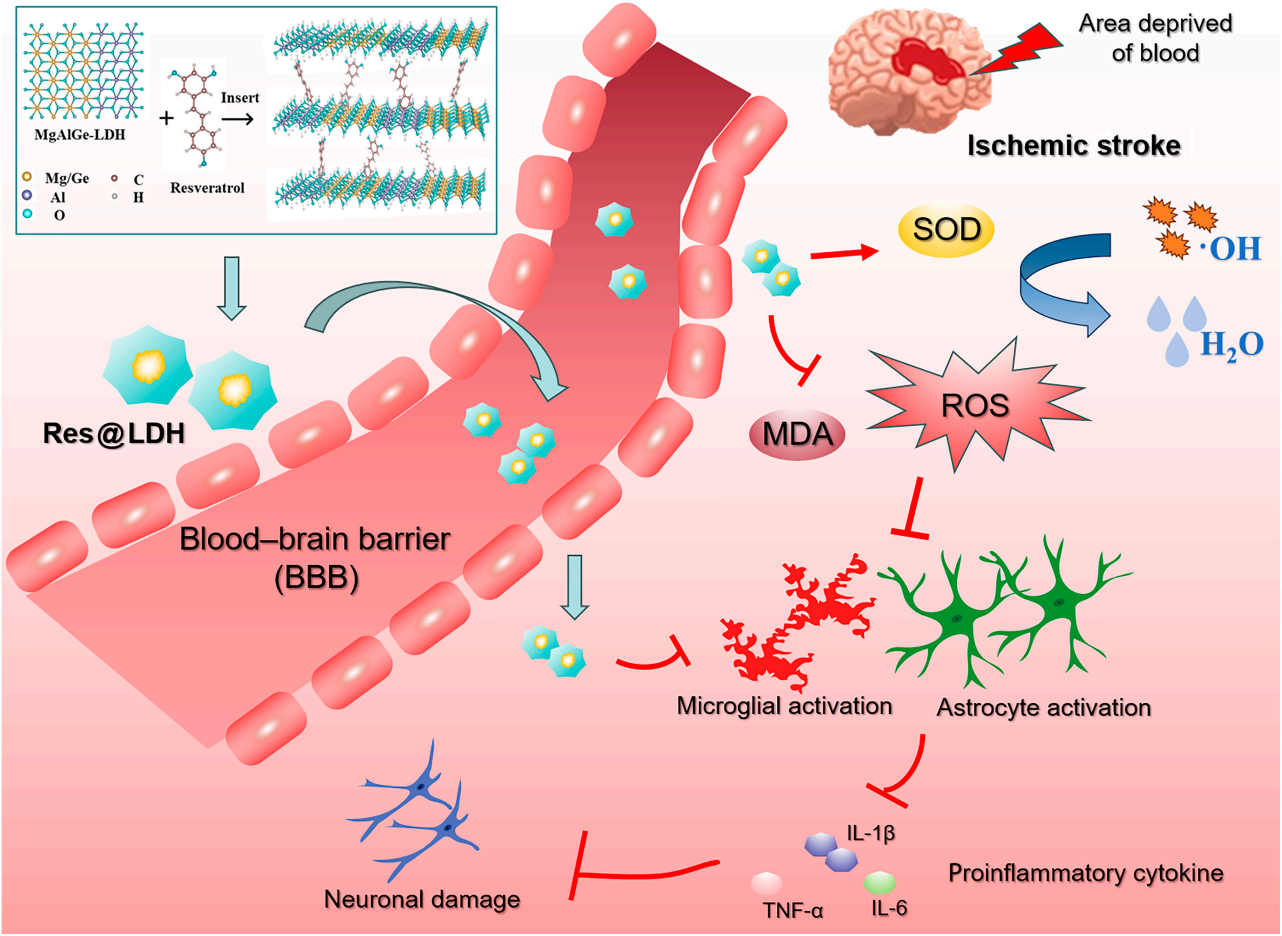
Schematic illustration of the regulation of ischemic stroke injury by Res@LDH treatment.

In this study, we present a stepwise synthesis of a nanoplatform for ischemic stroke therapy, as illustrated in Fig. 1. Initially, Ge is incorporated into MgAl-LDH via a simple one-step method, yielding MgAlGe-LDH (Ge-LDH). Subsequently, we synthesize a nanohybrid therapeutic agent utilizing Res and Ge-LDH nanomaterials as carriers (Res@LDH). Benefiting from the dual ROS scavenging effect of Res and Ge-LDH, the Res@LDH nanosystem exhibits excellent ROS scavenging efficiency and antioxidant activity, effectively mitigating oxidative stress and inflammation. Furthermore, Res@LDH enhances BBB permeability and brain tissue accumulation, surpassing the performance of Res alone. The abundant hydroxyl groups on the surface of Res@LDH contribute to its high biocompatibility and stability. Additionally, we evaluate the potential of Res@LDH in mice subjected to middle cerebral artery occlusion and reperfusion (MCAO-R) injury and HT22 cells experiencing H_2_O_2_-induced cytotoxicity. As anticipated, Res@LDH alleviates ischemic stroke injury by inhibiting oxidative stress damage and inflammation, associated with the reduction of microglial and astrocytic activation. Collectively, our study introduces a novel synthetic approach for nanotherapeutics utilizing Res and Ge-doped LDH, offering a promising neuroprotective strategy for treating ischemia–reperfusion injury.

## Materials and Methods

### Synthesis of LDH and Res@LDH

Nanosized LDH was synthesized by a typical chemical method. A mixed aqueous solution containing Mg(NO_3_)_2_·6H_2_O (0.03 M), Al(NO_3_)_3_·9H_2_O (0.01 M), and Ge(NO_3_)_3_·6H_2_O (0.2 g) was added to an aqueous solution containing NaNO_3_ (0.4 g) while stirring constantly at room temperature. Subsequently, 35 ml of 0.25 M NaOH was gradually introduced into the metal salt solution with continuous stirring at 80 °C. Following a 6-h reaction period, the LDH product was harvested via centrifugation at 10,000 rpm and washed 3 times with water followed by ethanol.

In the second stage, commercial Res and LDH were dispersed in deionized water. After stirring for 24 h in darkness, the Res@LDH product was collected by centrifugation at 10,000 rpm for 10 min.

### Animals

Male adult C57BL/6 mice aged 8 to 10 weeks old were obtained from Vital River (Beijing, China). The mice were randomly assigned to one of 4 groups (*n* = 33 per group): (a) the sham group (Sham), (b) the MCAO-R injury with saline group (MCAO), (c) the MCAO-R injury with LDH treatment group, and (d) the MCAO-R injury plus Res@LDH treatment group. Mice were euthanized 24 h after the surgical procedure. The animal studies were conducted following approval from the Ethics Committee of Laboratory Animal Welfare of Beijing Tongren Hospital (No. TRLAWEC2022-S123).

### Cell cultures

HT22 cells were cultured in Dulbecco’s modified Eagle medium supplemented with 10% fetal bovine serum, streptomycin, and penicillin at 37 °C in a 5% CO_2_ environment. The HT22 cells were subjected to H_2_O_2_-induced cytotoxicity for 4 h. Additionally, HT22 cells were exposed to various doses of LDH and Res@LDH for 24 h before being collected for subsequent analysis.

### Middle cerebral artery occlusion and reperfusion

Mice underwent MCAO-R injury using an intraluminal filament. Briefly, the mice were anesthetized with 2% pentobarbital, and their body temperature was maintained at 37 ± 0.5 °C. A suture was inserted from the left common carotid artery to the origin of the middle cerebral artery. After 1 h, reperfusion was initiated by removing the intraluminal suture. Subsequently, the mice received 20 mg/kg LDH or Res@LDH via tail vein injection at reperfusion. Physiological parameters, including rectal temperature, blood pressure, blood gas, and glucose levels, were monitored (Table S1).

### Garcia test

Neurological deficits were assessed 24 h postreperfusion by an observer blinded to the treatment groups using the Garcia test (*n* = 10) (Table S2). This test generates a composite score for each mouse by evaluating 6 distinct parameters: (a) spontaneous activity, (b) symmetry of limb movement, (c) forepaw outstretching, (d) cage climbing, (e) body proprioception, and (f) vibrissa reaction (Table S2). The total score ranges from a minimum of 3, indicating severe impairment, to a maximum of 18, indicating no impairment.

### Determination of BBB permeability

Following reperfusion, 4 ml/kg of 2% Evans blue was injected into the tail vein (*n* = 5 per group). Mice were euthanized 24 h after reperfusion. In the Evans blue extravasation assay, coronal sections were cut into 1-mm slices and photographed. For quantitative measurements, ischemic hemispheres were homogenized in *N*,*N*-dimethylformamide, followed by incubation and centrifugation. The supernatants were analyzed at 620 nm by spectrophotometry, and the change in BBB permeability was calculated.

### Cell Counting Kit-8 cell viability assay

HT22 cell viability in different groups was assessed with Cell Counting Kit-8 (CCK-8) assay (*n* = 10 per group). Briefly, 10 μl of CCK-8 solution was added to 100 μl of medium in each well of a 96-well plate and incubated for 2 h at 37 °C. Absorbance was measured at 450 nm using a microplate reader. Cell viability was calculated using the following equation: cell activity = (experimental treatment group − blank group)/(negative control group − blank group) × 100%.

### Dihydroethidium staining

ROS levels were detected using dihydroethidium (DHE) staining (*n* = 3 per group). Frozen coronal sections of mouse brains and HT22 cells plated on coverslips were incubated with the fluorescent dye DHE at 37 °C for 30 min in a humidified chamber and washed with phosphate-buffered saline 3 times. The sections were then observed via light microscopy.

### Immunofluorescence staining

The mice were anesthetized, and their brains were fixed via transcardial perfusion with 0.9% cold heparinized saline and 4% paraformaldehyde. Subsequently, the brains were sectioned into 5-μm-thick slices. These sections were utilized for DHE, hematoxylin–eosin (HE), Nissl, terminal deoxynucleotidyl transferase dUTP nick end labeling (TUNEL), and immunofluorescence staining.

For HE, Nissl, and TUNEL staining, sections were processed according to the manufacturer’s instructions, using HE, Nissl staining kit, and *In Situ* Cell Death Detection Kit, respectively (*n* = 5 per group). For immunofluorescence staining, the primary antibodies used included mouse anti-glial fibrillary acidic protein (anti-GFAP) antibodies (1:200; Cell Signaling Technology, USA) and rabbit anti-ionized calcium-binding adapter molecule 1 (anti-IBA1) antibodies (1:500; Wako, Japan). Following primary antibody incubation, the sections were examined under a fluorescence microscope.

### Statistical analysis

Statistical calculations were performed using GraphPad Prism 8.0. One-way analysis of variance (ANOVA) followed by Tukey’s post hoc test was conducted for comparisons among multiple groups. Garcia tests were presented as the median with the interquartile range and analyzed using 2-tailed Mann–Whitney U tests, while other values were presented as the mean ± standard deviation. *P* < 0.05 was considered statistically significant.

## Results

### Synthesis and characterization of Res@LDH

**Fig. 2.**
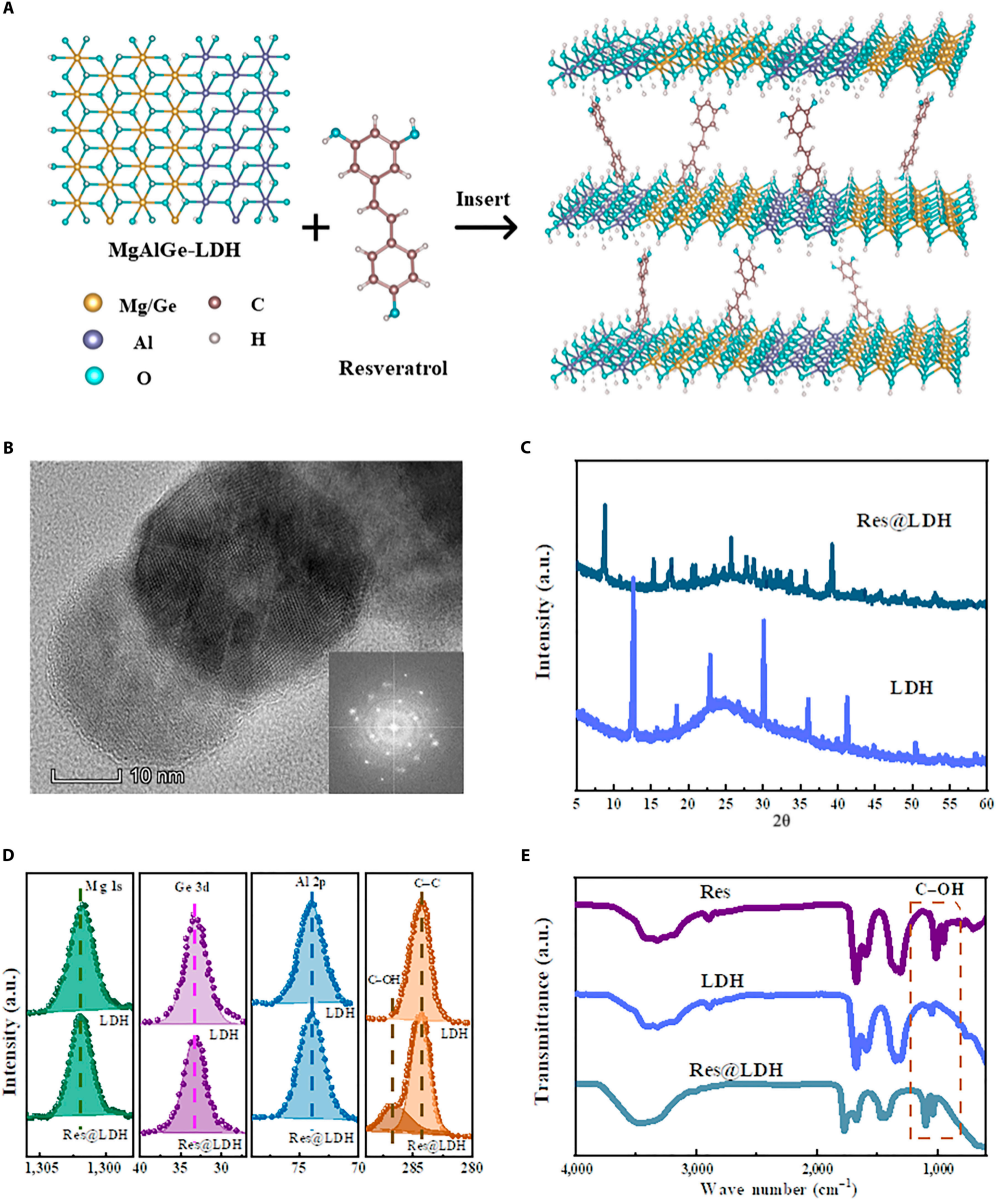
Structure, morphology, and chemical composition and characterization of Res@LDH. (A) Schematic illustration of the preparation of Res@LDH. (B) Transmission electron microscopy (TEM) images of Res@LDH. (C) X-ray diffraction (XRD) pattern of Res@LDH. (D) X-ray photoelectron spectroscopy (XPS) patterns of layered double hydroxide (LDH) and Res@LDH. (E) Fourier transform infrared (FTIR) spectra of resveratrol (Res), LDH, and Res@LDH.

The nanohybrid Res@LDH, with sizes up to 20 nm (Fig. S1), was successfully synthesized through an intercalation process using nanosized LDH as the precursor (detailed in Materials and Methods). Figure 2A and B illustrates the morphology of Res@LDH, with x-ray diffraction data in Fig. 2C indicating a complete replacement of reflections from the LDH precursors by a new set of reflections systematically shifted to lower angles. This shift suggests the incorporation of new molecular layers into the LDH layered structure. We performed scanning electron microscopy–energy-dispersive x-ray spectroscopy elemental mapping tests on the Res@LDH samples, and the results confirmed that their chemical composition of Ge-doped Mg/Al LDH is about Mg_0.72_Al_0.2_Ge_0.08_(OH)_2_ (Fig. S2).

X-ray photoelectron spectroscopy (XPS) analysis was conducted on LDH and Res@LDH powder samples to probe the species and oxidation states of the elements, as presented in Fig. 2D and Fig. S3. The survey spectra of LDH and Res@LDH revealed the presence of Mg, Al, Ge, and C–OH characteristic of the LDH sample (specifically, a MgAlGe-LDH) with Res molecules in the interlayers. Figure 2D further shows that the Mg 1s and Al 2p spectral peaks of pristine and hybrid materials have identical binding energies (1,302 and 74 eV) [37,38], indicating that the oxidation state of LDH remains unaltered upon reaction, with no evidence of charge transfer between the intercalated molecular layer and the host matrix. Additionally, a peak at 33 eV was attributed to Ge [39], confirming Ge doping within the LDH intralayer. In the LDH spectrum, a peak at 284 eV corresponds to the carbon paste used in the XPS measurements, while in Res@LDH, in addition to the peak representing the C–C bonds in the benzene ring of carbon gel and Res at 284 eV, there is a small peak at 287 eV, indicating the presence of C–OH bonds. Since only Res contains C–OH bonds in this reaction system, this serves as strong evidence for the successful incorporation of Res into the LDH layer [40].

Fourier transform infrared (FTIR) spectroscopy was utilized to identify the functional groups present on the hybrid materials, crucial for understanding their structure and intercalation process. Upon functionalization of LDH with Res, several characteristic peaks associated with Res molecules appeared in the FTIR spectra. The most prominent among these were the C–OH bonding-related modes in the 800- to 1,200-cm^−1^ region (Fig. 2E) [41]. The peaks in the range of 800 to 1,200 cm^−1^ experienced a redshift upon binding to LDH and Res, confirming the intercalation of Res molecules into the LDH interlayer. Furthermore, comparison of the spectra of Res@LDH with LDH revealed redshifted peaks at about 1,430 cm^−1^ (O–H bending) for Res@LDH, suggesting the formation of hydrogen bonds between Res and LDH [42].

### Synergistic effects of ROS scavenging by Res@LDH

**Fig. 3.**
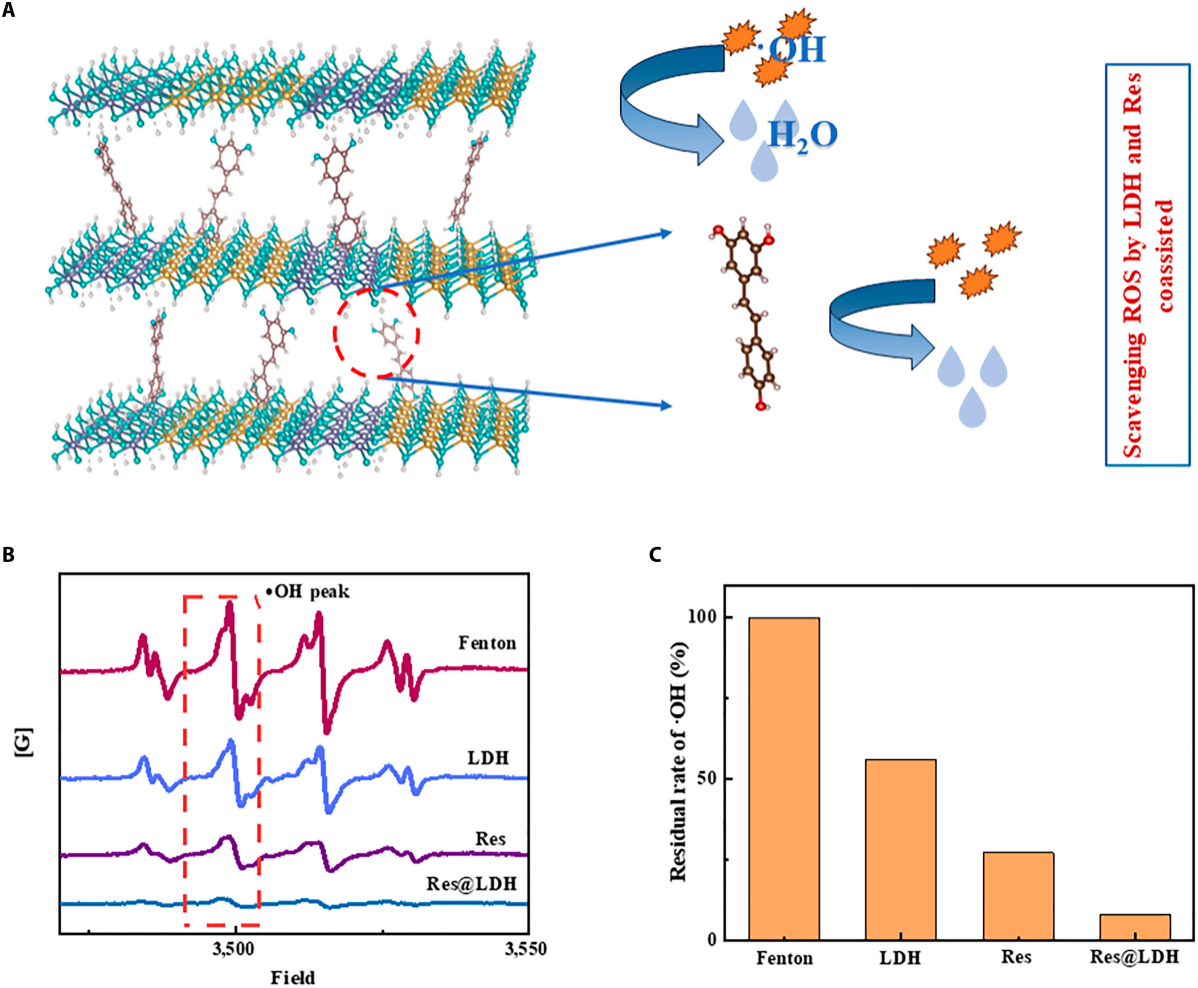
ROS scavenging activity of Res@LDH. (A) ROS scavenging mechanism of Res@LDH. (B) Electron paramagnetic resonance (EPR) spectra of DMPO-OH adducts formed in the presence of Res, LDH, and Res@LDH (all samples tested at the same concentration of 100 μg/ml). (C) •OH radicals were produced via the Fenton reaction using an Fe^2+^/H_2_O_2_ method and discovered with 5,5′-dimethylpyrroline-1-oxide (DMPO).

Given our objective to develop a treatment to mitigate ischemia–reperfusion injury, it was imperative to thoroughly investigate the ROS scavenging capabilities of the synthesized samples. In this work, partial substitution of Al for Mg in the LDH can introduce charges within the LDH layers. These charges mainly serve the following functions: (a) attracting the organic drug molecule Res, aiding its intercalation between the layers, and (b) allowing the synthesized Res@LDH nanoparticles to better adsorb OH radicals. Moreover, we report on germanium-doped LDH nanomaterials that facilitate more effective contact between the germanium catalyst and hydroxyl radicals (OH radicals) through adsorption and by crossing the BBB, thereby enhancing the catalytic decomposition of radicals by germanium. We employed an •OH scavenging assay, utilizing electron paramagnetic resonance (EPR) spectroscopy for quantification. In this assay, •OH radicals were generated by the Fe^2+^/H_2_O_2_ system and quantified using 5,5′-dimethylpyrroline-1-oxide (DMPO) as the trap (Fig. 3A).

In the absence of a radical scavenger, the Fenton reaction generated strong EPR signals for the DMPO-OH adduct (Fig. 3B and C). The inclusion of Ge-LDH into the system reduced the intensity of the EPR signal for the DMPO-OH adduct by about 50%, indicating the ROS scavenging ability of LDH. It is noteworthy that typical LDH-type materials lack such capability, suggesting that the incorporation of Ge ions into the lattice may confer this ability. In fact, in Fig. 3, we tested the ROS scavenging rate of Mg/Al-LDH containing Ge and found that it can remove about 50% of OH radicals. The ROS scavenging rate of Mg/Al-LDH without Ge has been reported in the previous literature; according to the report by Wang et al. [25], the ROS scavenging rate of pure Mg/Al-LDH is only about 10%.

Res showed a marked reduction in the EPR signal, consistent with its known ROS scavenging ability. Notably, the EPR signal of Res@LDH was very weak, indicating that the assembled nanohybrid possessed excellent ROS (•OH) scavenging properties. These results demonstrate that both LDH and Res individually possess the ability to scavenge ROS, specifically hydroxyl radicals (•OH), and the interactions between Res and Ge ions in the LDH nanosheets likely contribute to the enhanced ROS scavenging activity observed in Res@LDH.

### Res@LDH attenuated cell damage and inhibited oxidative stress

**Fig. 4.**
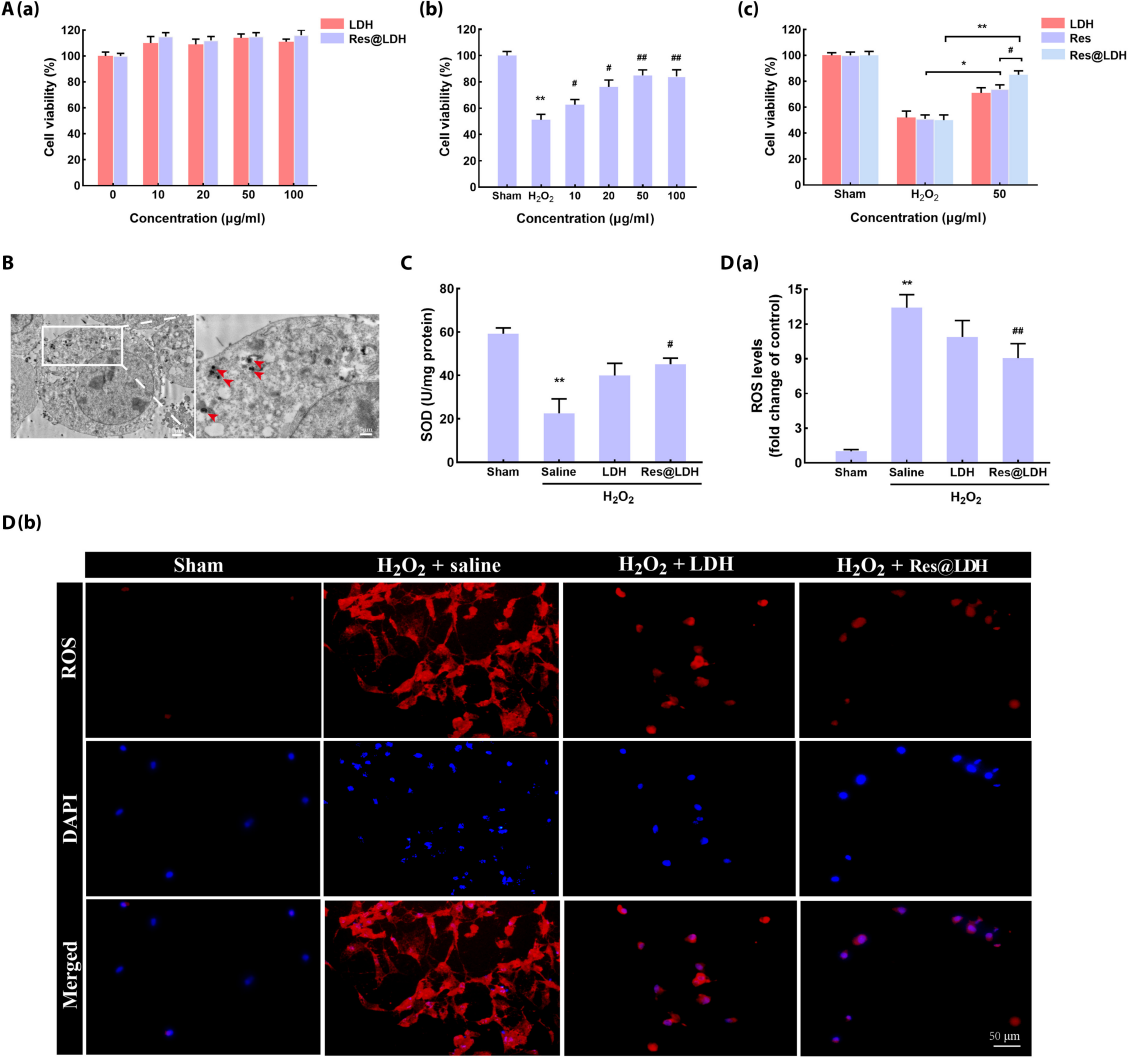
ROS scavenging and endocytosis of Res@LDH in vitro. (A) (a) HT22 cell viability after incubation with Res@LDH for 24 h. *n* = 10 each. (b) Cell viability of HT22 cells incubated with H_2_O_2_ and various concentrations of Res@LDH for 24 h. ^**^P < 0.01 vs. Sham group; ^#^*P* < 0.05 and ^##^*P* < 0.01 vs. H_2_O_2_ group. *n* = 10 each. (c) The cell viability of HT22 cells incubated with H_2_O_2_ and 50 μg/ml LDH or Res@LDH. ^*^P < 0.05 vs. H_2_O_2_-Res group; ^**^P < 0.01 vs. H_2_O_2_-Res@LDH group; ^#^*P* < 0.05 vs. 50-Res group. *n* = 10 each. (B) Representative TEM images of Res@LDH internalized in HT22 cells. *n* = 3. (C) Superoxide dismutase (SOD) levels in HT22 cells. ^**^P < 0.01 vs. Sham group; ^#^*P* < 0.05 vs. H_2_O_2_–saline group. *n* = 5 each. (D) (a) ROS levels in HT22 cells. (b) Representative dihydroethidium (DHE) staining showing the ROS levels in HT22 cells incubated with H_2_O_2_ and 50 μg/ml LDH or Res@LDH for 24 h. Scale bars, 50 μm. ^**^P < 0.01 vs. Sham group; ^##^*P* < 0.01 vs. H_2_O_2_–saline group. *n* = 3 each.

To further elucidate the excellent ROS scavenging properties of the nanocomposites, we conducted in vitro experiments using mouse hippocampal neuronal cell lines, HT22 cells. Firstly, we employed a CCK-8 cell viability assay to assess HT22 cell viability after 24-h treatment with LDH and Res@LDH at increasing doses. As depicted in Fig. 4A (a), HT22 cell viability remained above 95%, indicating the remarkable biocompatibility of both LDH and Res@LDH, even at a high concentration of 100 μg/ml.

Subsequently, we investigated the antioxidant ability of Res@LDH in a H_2_O_2_-induced cytotoxicity assay with HT22 cells, a cell model for ischemic stroke injury. As illustrated in Fig. 4A (b), H_2_O_2_ injury decreased HT22 cell viability to 51.2%, which could be rescued after 24-h treatment with Res@LDH, even at a low concentration of 10 μg/ml (62.8%), increasing in a dose-dependent manner (85% at 50 μg/ml). Hence, we selected 50 μg/ml Res@LDH for subsequent in vitro experiments. Additionally, both LDH alone (50 μg/ml) and free Res alone (50 μg/ml) enhanced HT22 cell viability following H_2_O_2_-induced injury. Notably, Res@LDH resulted in a greater increase in cell viability compared to free Res, confirming the potential synergistic effect between LDH and Res (Fig. 4A (c)).

Then, we employed transmission electron microscopy to investigate HT22 cell morphology after Res@LDH treatment for 24 h, showing effective uptake, internalization, and accumulation in lysosomes (Fig. 4B). Moreover, the level of superoxide dismutase (SOD) was decreased in HT22 cells after H_2_O_2_ injury, but the SOD levels were substantly increased with treatment with LDH and Res@LDH, respectively (Fig. 4C). DHE staining was utilized to examine ROS levels in HT22 cells after H_2_O_2_ injury (Fig. 4D). As expected, H_2_O_2_ injury led to marked higher levels of ROS to the vehicle (saline) control, while LDH treatment (H_2_O_2_ + LDH group) exhibited a slight ROS scavenging ability (Fig. 4D). Treatment with Res@LDH (H_2_O_2_ + Res@LDH group) revealed a strong free radical scavenging in HT22 cells.

### Res@LDH attenuated cerebral ischemic injury and BBB damage

**Fig. 5.**
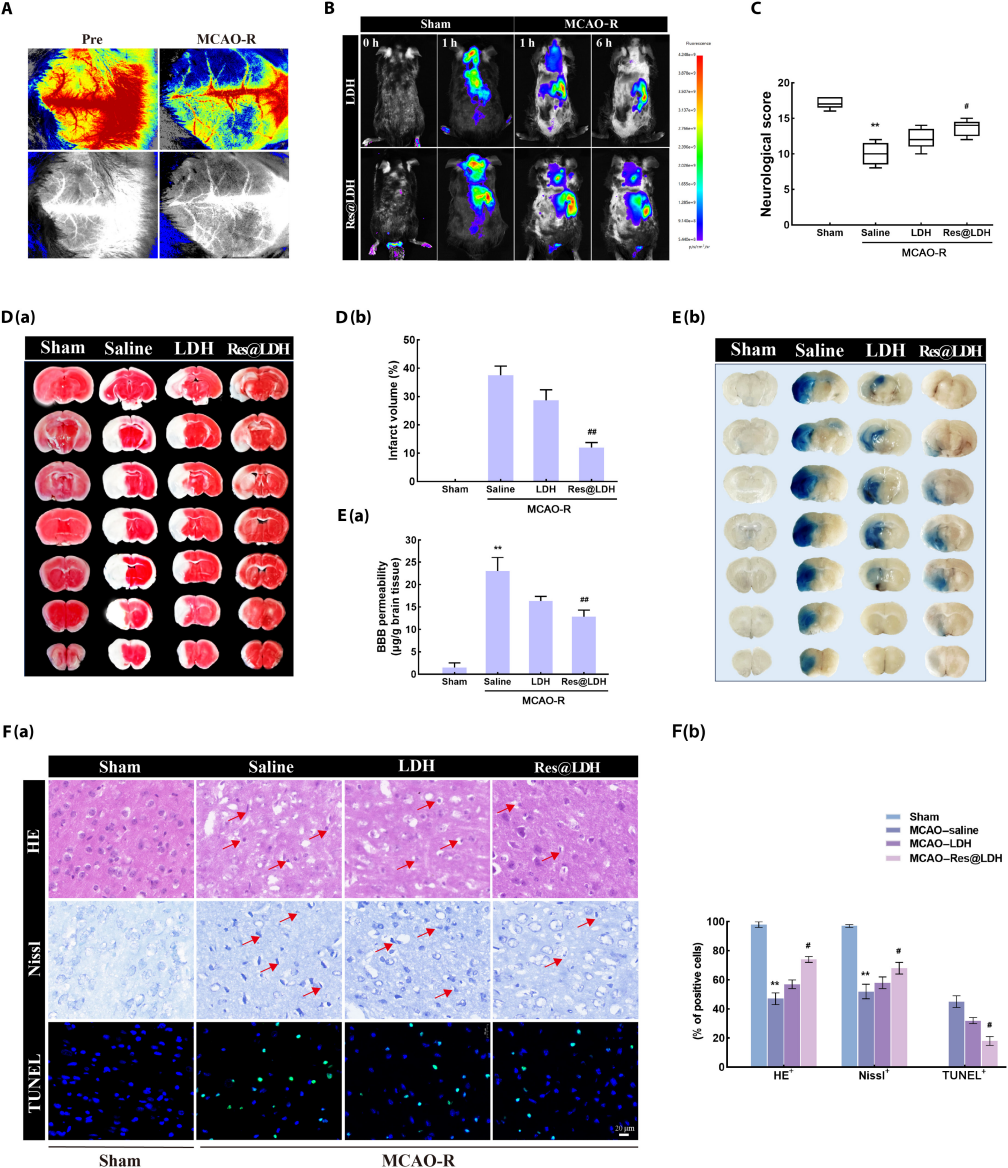
Neuroprotection of Res@LDH treatment against ischemic stroke injury. (A) Cerebral blood flow (CBF) conducted by laser speckle imaging along middle cerebral artery occlusion (MCAO)/reperfusion surgeries. *n* = 3 each. (B) In vivo imaging of indocyanine green (ICG)-labeled LDH and Res@LDH in healthy and MCAO mouse models at different time points. *n* = 3 each. (C) Neurological deficit score evaluated 24 h after reperfusion. ^**^P < 0.01 vs. Sham group; ^#^*P* < 0.05 vs. MCAO–saline group. *n* = 10 each. (D) (a) Representative photographs of brain slices showing the infarct volume 24 h after reperfusion. (b) Statistical analysis of infarction volume in different groups. ^##^*P* < 0.01 vs. MCAO–saline group. *n* = 10 each. (E) (a) Quantitative assay of Evans blue leakage in different groups 24 h after reperfusion. ^**^P < 0.01 vs. Sham group; ^##^*P* < 0.01 vs. MCAO–saline group. (b) Representative photographs of Evans blue extravasation in the brains in different groups 24 h after reperfusion. *n* = 5 each. (F) (a) Hematoxylin–eosin (HE) staining showing cell morphologic changes, Nissl staining showing neuronal morphological changes, and terminal deoxynucleotidyl transferase dUTP nick end labeling (TUNEL) staining showing neuronal apoptosis in the ischemic penumbra 24 h after reperfusion. Scale bar = 20 μm. (b) Statistical analysis of complete cells (HE^+^), intact neurons (Nissl^+^), and neuronal apoptosis (TUNEL^+^) in the ischemic penumbra. ^**^P < 0.01 vs. Sham group; ^#^*P* < 0.05 vs. MCAO–saline group. *n* = 5 each.

In our in vivo experiments, we utilized an MCAO-R mouse model to investigate the neuroprotective effects of Res@LDH. To visually observe the process of ischemia–reperfusion, we measured CBF using laser speckle imaging. The cortical CBF value decreased to 15% of the preischemic value during occlusion and then recovered to 70% of the baseline after reperfusion, confirming the successful establishment of the MCAO-R mouse model (Fig. 5A).

To assess the ability of LDH and Res@LDH to reach the brain and penetrate the BBB, we investigated their in vivo biodistribution in normal mice and MCAO-R model mice. Through slight modifications of LDH and Res@LDH, we synthesized photosensitizer indocyanine green (ICG)-labeled nanodrugs to track the distribution of these materials in the brain. Figure 5B demonstrates that both LDH and Res@LDH reached the brain site 1 h after intravenous injection in normal mice. Similarly, strong fluorescence signals were detected in the brain site for LDH and Res@LDH treatment after MCAO-R injury (1 h after administration). In MCAO-R mice, 6 h after administration, no obvious fluorescence intensity was observed in the brain site of LDH-treated animals, while a strong fluorescent signal was still present in the Res@LDH-treated group. These findings suggest that Res@LDH can penetrate the BBB and accumulate in the brain in healthy and MCAO-R-injured mice.

Res@LDH administration caused no changes in the levels of alanine transaminase, aspartate transaminase, serum creatinine, and blood urea nitrogen (Fig. S4), indicating no liver and kidney function damage. This positive tolerability and safety drug profile in vivo enabled further investigation of Res@LDH neuroprotective efficacy in ischemic stroke injury in mice through neurological scores and 2,3,5-triphenyltetrazolium chloride staining to determine brain injury (Fig. 5C and D). The standard of neurological scores is shown in Table S2. As expected, MCAO-R injury notably worsened neurological scores; however, these were improved by LDH and Res@LDH treatment. Additionally, the neurological deficit scores of MCAO-R mice after Res@LDH treatment were higher than those after LDH treatment alone (Fig. 5C). Moreover, the infarct volume in the MCAO–saline group was 37.5% ± 3.0%, while after treatment with LDH and Res@LDH, the infarct volume decreased to 28.7% ± 3.4% and 12.3% ± 1.6%, respectively (Fig. 5D).

We next measured BBB permeability among the 4 groups by Evans blue staining (Fig. 5E). Evans blue staining was markedly increased in the MCAO injured group, but it was lower with treatment with LDH and Res@LDH, respectively. Res@LDH treatment showed the lowest Evans blue staining after MCAO-R injury.

Furthermore, we performed HE, Nissl, and TUNEL staining to assess neuronal damage and survival in the ischemic penumbra following MCAO-R injury. HE-stained cells (HE^+^) had intact nucleoli, compact structures, and clear outlines (Fig. 5F). The proportion of HE^+^ cells decreased significantly (from 98.3% ± 2.6% in Sham) to 47.2% ± 4.0% following MCAO-R injury. Treatment with LDH and Res@LDH increased the proportions of HE^+^ cells to 57.5% ± 3.4% and 74.1% ± 2.2%, respectively. Additionally, the proportions of Nissl^+^ cells were significantly decreased to 52.3% ± 5.1% in the MCAO–saline group and again was rescued following LDH and Res@LDH treatment (58.8% ± 4.5% and 68.7% ± 4.6%, respectively). Furthermore, Res@LDH exhibited the highest proportions of HE^+^ and Nissl^+^ cells following MCAO-R injury among the 4 groups. TUNEL-stained cells (TUNEL^+^) represented apoptosis. Compared with the Sham group, TUNEL^+^ cells increased significantly to 45.8% ± 4.4% following MCAO-R injury. However, treatment with LDH and Res@LDH reduced neuronal apoptosis to 32.2% ± 2.5% and 18.8% ± 3.6%, respectively.

### Res@LDH inhibited oxidative stress in the ischemia penumbra

**Fig. 6.**
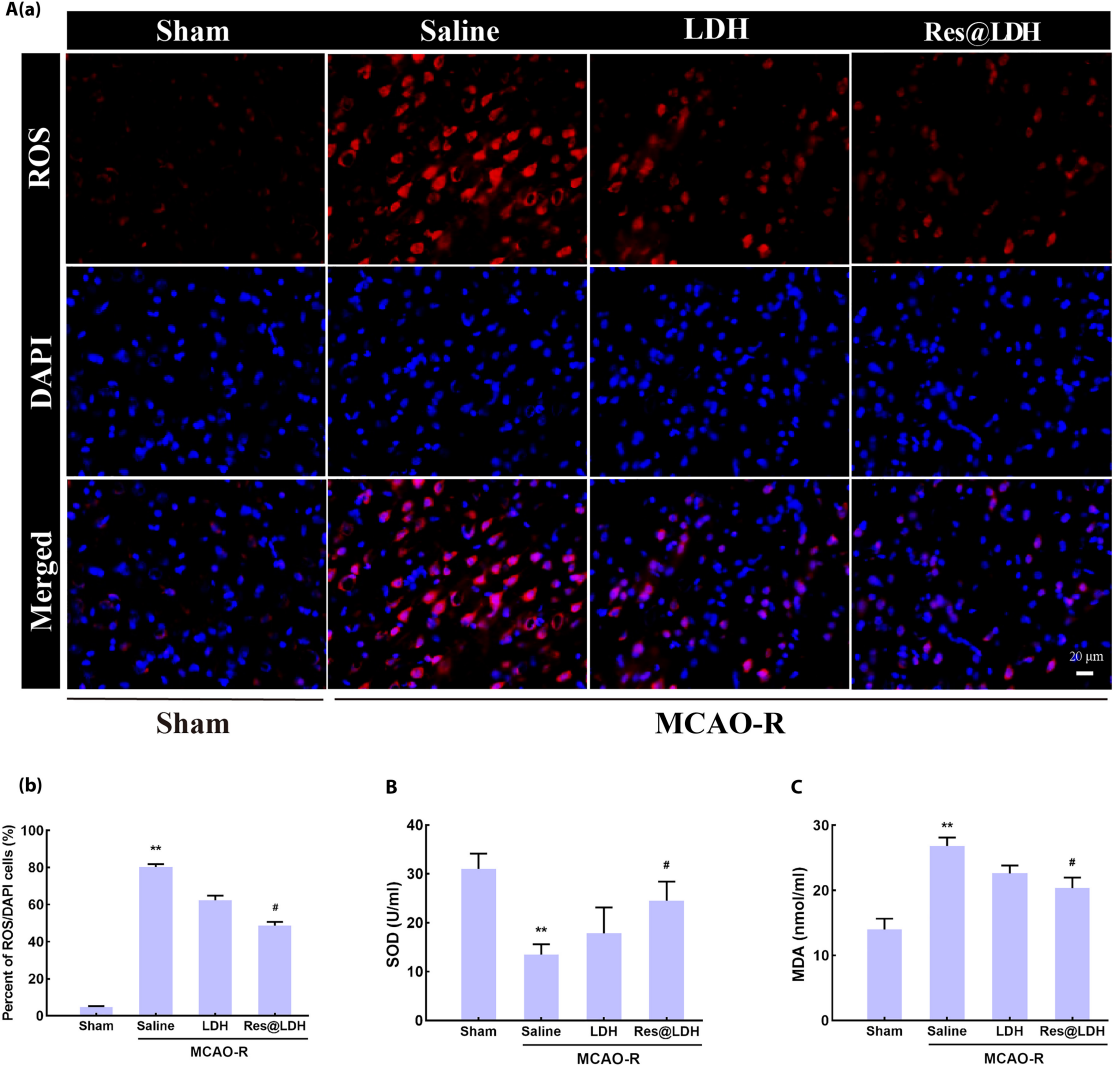
Res@LDH treatment attenuated oxidative stress in the ischemic penumbra. (A) (a) Representative DHE staining showing the ROS levels in the penumbra after reperfusion. Scale bar = 20 μm. (b) Statistical analysis of the percentage of ROS-positive cells in the penumbra. ^**^P < 0.01 vs. Sham group; ^#^*P* < 0.05 vs. MCAO–saline group. *n* = 3 each. (B) The levels of SOD in the serum in different groups. ^**^P < 0.01 vs. Sham group; ^#^*P* < 0.05 vs. MCAO–saline group. *n* = 5 each. (C) The levels of malondialdehyde (MDA) in the serum in different groups. ^**^P < 0.01 vs. Sham group; ^#^*P* < 0.05 vs. MCAO–saline group. *n* = 5 each.

Ischemic stroke injury is highly vulnerable to high levels of ROS-induced oxidative stress damage, owing to its elevated aerobic metabolism and blood perfusion [43]. To assess the ROS scavenging properties of the nanocomposites in vivo, we employed DHE staining to examine ROS levels after reperfusion in the ischemic penumbra in the 4 groups. The percentage of ROS-positive cells was markedly increased in the MCAO–saline group compared with that in the Sham group (Fig. 6A). However, treatment with LDH or Res@LDH resulted in a decrease in the levels of ROS-positive cells. Importantly, the levels of ROS-positive cells in the MCAO-Res@LDH group were dramatically reduced compared to those in the MCAO–saline group.

To further verify the antioxidative effect of Res@LDH, we measured the activity of SOD, the major antioxidant enzyme involved in protecting nervous tissue from oxidative stress, and the levels of malondialdehyde (MDA), which are byproducts of lipid peroxidation. The levels of SOD were 31.0 ± 2.7 U/ml in the Sham group, whereas they were significantly decreased in the MCAO model (13.5 ± 1.5 U/ml). Treatment with LDH or Res@LDH increased the levels of SOD to 17.9 ± 4.7 and 24.6 ± 3.6 U/ml, respectively (Fig. 6B). Similarly, the levels of MDA were 14.0 ± 1.4 U/ml in the Sham group, and they were significantly increased in MCAO injured animals (26.8 ± 1.2 U/ml). Treatment with LDH decreased the level of MDA to 22.6 ± 1.1 U/ml, and these were significantly decreased after Res@LDH treatment (20.4 ± 1.5 U/ml), once more exhibiting the highest levels of SOD and the lowest levels of MDA among the groups (Fig. 6C).

### Res@LDH inhibited inflammation mediated by the activation of astrocytes and microglia

**Fig. 7.**
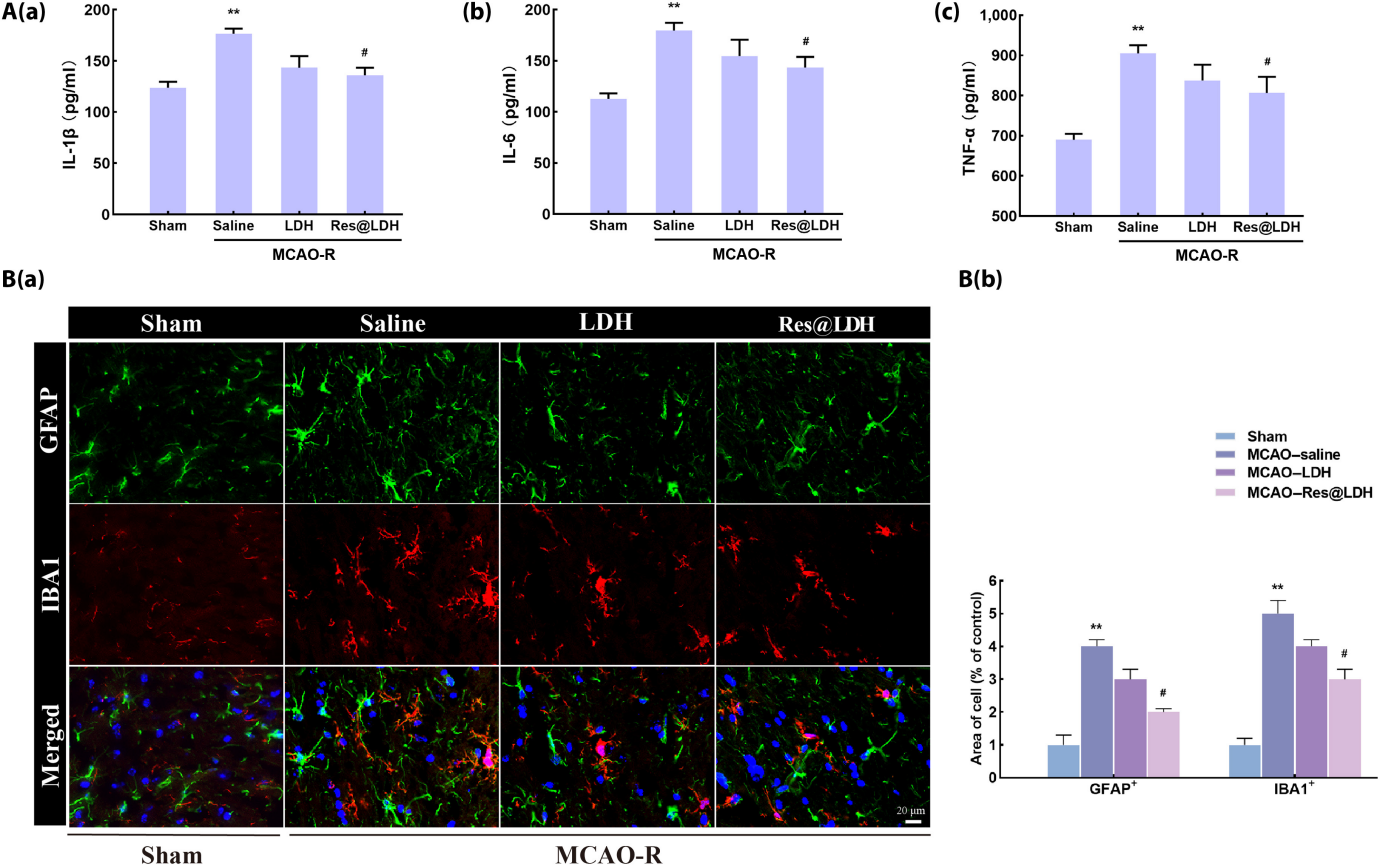
Res@LDH treatment relieved astrocyte- and microglia-mediated inflammatory activation. (A) Interleukin-1 beta (IL-1β) (a), interleukin-6 (IL-6) (b), and tumor necrosis factor alpha (TNF-α) (c) levels in the serum in different groups. ^**^P < 0.01 vs. Sham group; ^#^*P* < 0.05 vs. MCAO–saline group. *n* = 5 each. (B) (a) Representative immunofluorescence images showing astrocyte (GFAP^+^) and microglia (IBA1^+^) morphology in the ischemic penumbra. Scale bar = 20 μm. (b) Statistical analysis of the percentage of GFAP-positive and IBA1-positive cells in the penumbra. ^**^P < 0.01 vs. Sham group; ^#^*P* < 0.05 vs. MCAO–saline group. *n* = 5 each.

Neuroinflammation stands as a pivotal mechanism in ischemic stroke injury. Hence, we assessed the expression of proinflammatory cytokines (interleukin-1 beta [IL-1β], interleukin- 6 [IL-6], and tumor necrosis factor alpha [TNF-α]) 24 h after reperfusion. As depicted in Fig. 7A, the level of IL-1β was 123.8 ± 5.3 pg/ml in the Sham group, escalating significantly to 176.5 ± 4.6 pg/ml in the MCAO–saline group. Post-LDH and post-Res@LDH treatment, the levels of IL-1β diminished to 143.4 ± 10.9 and 135.9 ± 7.0 pg/ml, respectively (Fig. 7A (a)). Similarly, the levels of IL-6 were 112.7 ± 4.9 pg/ml in the Sham group and rose to 179.4 ± 6.9 pg/ml in the MCAO–saline group. Following LDH treatment, the level decreased to 154.6 ± 15.2 pg/ml, while Res@LDH treatment notably lowered it to 143.4 ± 9.8 pg/ml (Fig. 7A, (b)). Regarding TNF-α, the level was 690.0 ± 13.4 pg/ml in the Sham group, escalating significantly to 905.5 ± 16.4 pg/ml following ischemic stroke injury. LDH treatment reduced the level to 838.0 ± 17.4 pg/ml, and Res@LDH treatment further decreased it to 807.0 ± 18.5 pg/ml (Fig. 7A, (c)).

Finally, we investigated the role of Res@LDH in astrocytic and microglial activation and measured its activation in the ischemic penumbra across the 4 groups. Compared with the Sham group, the astrocytes (GFAP positive) and microglia (IBA1 positive) in the MCAO group exhibited heightened activation, evident from short, coarse cytoplasmic processes, enlarged somas, and hypertrophic processes (Fig. 7B). However, LDH and Res@LDH treatment attenuated astrocytic and microglial activation induced by MCAO-R injury. Notably, compared with the MCAO group, Res@LDH treatment notably alleviated the activation of astrocytes and microglia.

## Discussion

The utilization of nanomaterials in stroke therapy heralds a promising frontier in neurology. Nanoparticles present unique advantages, including precise targeting, improved drug delivery, and regulated release kinetics [44,45]. In the context of ischemic stroke, nanomaterials play an important role in mitigating neuronal damage, oxidative stress, and inflammation, thereby holding the potential to enhance patient outcomes [44–46].

LDHs possess notable characteristics, including a compact size, a well-defined structure, biocompatibility, and cost-effective production methods [25–28]. The antioxidant properties of LDHs are attained through the integration of diverse metal ions like copper, manganese, and gadolinium into their structure [25–31]. Here, we focused on germanium (Ge)-doped LDHs as potential candidates for alleviating ROS in ischemic stroke injury.

Res, due to its antioxidant and anti-inflammatory properties, has garnered substantially attention for its neuroprotective effects, making it as a promising candidate for ischemic stroke therapy [32–34]. However, its limited bioavailability and poor solubility have hampered its clinical translation. Considering these, we present Res@LDH, comprising Ge-containing layered double hydroxide nanosheets (Ge-LDH) as a drug nanocarrier and Res as a neuroprotective agent to explore the role of Res@LDH in ischemic stroke injury. That is, the hybrid material Res@LDH was successfully synthesized through a conventional chemical process, resulting in nanosized particles (~20 nm). Our EPR tests revealed that both Ge-doped LDH and the conventional drug Res exhibit excellent ROS scavenging properties. When combined, they form a nanohybrid therapeutic agent, Res@LDH, which has remarkable synergistic ROS scavenging efficiency (approximately 90%). In our study, we confirmed the incorporation of new molecular layers into the LDH layered structure, and the Res@LDH material exhibits a synergistic interaction between the drug molecules and the nanocarrier, promoting the quenching of hydroxyl radicals (•OH) and suppressing the generation of ROS. This synergy holds markedly promise for mitigating ischemia–reperfusion injury, prompting us for further exploration in vitro models.

Moreover, in vivo biodistribution experiments demonstrated that Res@LDH possesses superior BBB permeability, enhancing its accumulation in brain tissue. We observed substantially accumulation of Res@LDH in the brain of MCAO-R-injured mice at 1 and 6 h postadministration, indicating rapid penetration across the BBB and Res@LDH accumulation in the brain. Furthermore, Res@LDH levels were decreased at 24 h postadministration (Fig. S5), showing that the nanohybrid remained in the brain tissue for an extended period but began to reduce over time. Thus, we acknowledge the need for future studies to include longer time points for a more thorough evaluation of Res@LDH’s retention in the brain. The penetration and accumulation of Res@LDH in the brain may be attributed to several factors: (a) Nanoparticle size and surface modification: The small size and surface characteristics of Res@LDH nanoparticles facilitate their transport across the BBB. Surface modifications, such as coating with specific ligands or polymers, can enhance BBB penetration. (b) Enhanced permeability effect: In the case of MCAO-R-damaged mice, the BBB may be compromised, leading to an enhanced permeability and retention effect, allowing easier access for Res@LDH nanoparticles. (c) Lipophilicity: The conjugation with LDH may enhance the lipophilicity of Res, which is beneficial for crossing the lipid-rich environment of the BBB. (d) Stability and protection: The LDH carrier protects Res from degradation in the bloodstream, ensuring that a higher concentration of the active compound reaches the BBB. These mechanisms collectively improve the delivery and therapeutic efficacy of Res in the brain, surpassing the limitations of free Res. Next, in mice with middle cerebral artery occlusion, Res@LDH notably alleviated neuronal injury and infarct volume in the ischemic penumbra, reflecting the excellent neuroprotection efficacy. In H_2_O_2_-induced cytotoxicity in HT22 cells, Res@LDH treatment markedly attenuated cell damage. Our cellular and animal experiments provided valuable insights into the efficacy of the synthesized nanomaterials in alleviating stroke-induced damage.

Clearing ROS emerges as a pivotal therapeutic strategy for addressing ischemic stroke injury [11–13]. The ROS scavenging abilities of the synthesized nanomaterials were assessed in both cellular and animal models of ischemic stroke. Our findings revealed that Res@LDH treatment effectively alleviated ischemic-stroke-injury-induced oxidative stress, potentially providing synergistic therapeutic benefits of LDH and Res and offering a promising new strategy for mitigating oxidative-stress-induced cell damage in ischemic stroke.

After ischemic stroke injury, microglia and astrocytes undergo excessive activation, characterized by large cell bodies and hypertrophic processes [19,47]. Subsequently, these activated microglia and astrocytes release high levels of inflammatory cytokines, exacerbating neuroinflammation and ischemic stroke injury. Thus, inhibiting the activation of microglia and astrocytes could markedly alleviate ischemic stroke injury [48]. Several studies have shown that Res exerts anti-inflammatory effects in microglia and astrocytes by inhibiting proinflammatory cytokines [49,50]. Therefore, we investigated the role of Res@LDH in astrocytic and microglial activation and measured their activation in the ischemic penumbra across the 4 groups. Our results suggest that Res@LDH effectively inhibits the activation of astrocytes and microglia during ischemic stroke injury, thereby mitigating inflammatory damage. By inhibiting the overactivation of these glial cells, the nanomaterials effectively attenuated neuroinflammation, thereby preserving neuronal function and integrity. The overall trend across multiple parameters—such as enhanced neuroprotection, improved BBB integrity, superior antioxidant activity, and stronger anti-inflammatory effects—supports the synergistic action of Res and LDH, suggesting that Res@LDH provides a superior neuroprotective effect compared to LDH alone. The schematic illustration of the regulation of ischemic stroke injury by Res@LDH treatment is shown in Fig. 1.

 Despite the promising results obtained with Res@LDH, several limitations must be acknowledged. First, the in vivo studies were conducted over a relatively short period, which may not fully capture the long-term effects and potential chronic toxicity of Res@LDH. Future studies should extend the observation period to evaluate the prolonged efficacy and safety of the nanohybrid therapeutic. Second, while our study demonstrated enhanced BBB permeability and neuroprotection, the exact mechanisms by which Res@LDH interacts with and modulates cellular and molecular pathways in ischemic stroke remain to be fully elucidated. Third, further mechanistic studies are needed to explore how Res@LDH affects signaling pathways and cellular responses in more detail. Additionally, the study was limited to specific animal models and cell lines, which may not completely represent the variability seen in human ischemic stroke cases. Future research should include a broader range of models and patient-derived cells to validate the findings across different biological contexts. Lastly, the scalability and reproducibility of the Res@LDH synthesis process need to be addressed to ensure that it can be translated into clinical applications. Investigations into optimizing the production and evaluating the clinical feasibility of Res@LDH are essential next steps. Addressing these limitations will provide a more comprehensive understanding of Res@LDH’s therapeutic potential and pave the way for its development as a viable treatment for ischemic stroke.

## Conclusion

This study introduces a novel nanotherapeutic approach utilizing Res and Ge-LDH, providing a promising neuroprotective strategy for ischemia–reperfusion injury. By targeting oxidative stress, inflammation, and glial cell activation, the synergistic action of Res@LDH highlights its potential as a therapeutic intervention for ischemic strokes, offering new avenues for the treatment of this debilitating condition.

## Ethics Approval

The animal studies were conducted following approval from the Ethics Committee of Laboratory Animal Welfare of Beijing Tongren Hospital (No. TRLAWEC2022-S123).

## Data Availability

The datasets used and/or analyzed during the current study are available from the corresponding authors on reasonable request.
