# Supplementary material for: Res@LDH: A Novel Nanohybrid Therapeutic for Ischemia–Reperfusion Injury with Dual Reactive Oxygen Species Scavenging Efficiency
Source: Biomater Res. 2024 Dec 3;28:0108. doi: 10.34133/bmr.0108 (PMC11612122; doi:10.34133/bmr.0108)
Supplement: Supplementary 1 — Figs. S1 to S5 Tables S1 and S2 [file bmr.0108.f1.zip › Supplementary Figures.docx]

Supplementary Figure S1. TEM photograph of Ge-LDH.

Supplementary Figure S2. SEM-EDS elementary mapping for Ge-LDH.

Supplementary Figure S3. XPS survey spectra of LDH and hybrid Res@LDH.

Supplementary Figure S4. Blood panel analysis A. ALT levels in the above groups. n = 7 per group. B. AST levels in the above groups. n = 7 per group. C. SCr levels in the above groups. n = 7 per group. D. BUN levels in the above groups. n = 7 per group. ALT: alanine transaminase; AST: aspartate transaminase; SCr: Serum creatinine; BUN: Blood urea nitrogen.

Supplementary Figure S5. In vivo imaging of ICG-labelled Res@LDH in MCAO mice model at different time points. n = 3 each.
