# Supplementary material for: Res@LDH: A Novel Nanohybrid Therapeutic for Ischemia–Reperfusion Injury with Dual Reactive Oxygen Species Scavenging Efficiency
Source: Biomater Res. 2024 Dec 3;28:0108. doi: 10.34133/bmr.0108 (PMC11612122; doi:10.34133/bmr.0108)
Supplement: Supplementary 1 — Figs. S1 to S5 Tables S1 and S2 [file bmr.0108.f1.zip › Supplementary Table S1.docx]

| Group | | MABP  (mmHg) | Temp  (oC) | Glu  （dl/ml） | Hct  (%) | pH | pO_2_  (mmHg) | pCO2  (mmHg) |
| --- | --- | --- | --- | --- | --- | --- | --- | --- |
| MCAO/R-Saline | pre | 67±3 | 37±0.3 | 182±11 | 27±2 | 7.4±0.1 | 136±4 | 45±2 |
|  | during | 71±2 | 37±0.1 | 187±10 | 32±3 | 7.4±0.1 | 138±3 | 52±3 |
|  | post | 68±1 | 37±0.2 | 172±11 | 28±3 | 7.4±0.1 | 142±5 | 51±4 |
| MCAO/R-LDH | pre | 65±1 | 37±0.3 | 167±12 | 28±2 | 7.4±0.1 | 134±4 | 47±5 |
|  | during | 71±3 | 37±0.2 | 165±13 | 32±4 | 7.4±0.1 | 136±5 | 51±3 |
|  | post | 69±1 | 37±0.1 | 161±11 | 27±2 | 7.4±0.1 | 142±5 | 48±6 |
| MCAO/R-Res@LDH | pre | 72±2 | 37±0.1 | 181±13 | 32±3 | 7.4±0.1 | 142±3 | 48±4 |
|  | during | 67±3 | 37±0.2 | 158±13 | 31±2 | 7.4±0.1 | 139±5 | 52±4 |
|  | post | 68±1 | 37±0.3 | 177±12 | 28±3 | 7.4±0.1 | 135±6 | 44±3 |

**Supplementary Table S1.** The physiological parameters of mice.
