# Supplementary material for: Res@LDH: A Novel Nanohybrid Therapeutic for Ischemia–Reperfusion Injury with Dual Reactive Oxygen Species Scavenging Efficiency
Source: Biomater Res. 2024 Dec 3;28:0108. doi: 10.34133/bmr.0108 (PMC11612122; doi:10.34133/bmr.0108)
Supplement: Supplementary 1 — Figs. S1 to S5 Tables S1 and S2 [file bmr.0108.f1.zip › Supplementary Table S2.docx]

| Test | Score | | | |
| --- | --- | --- | --- | --- |
|  | 0 | 1 | 2 | 3 |
| Spontaneous activity (in cage for 5 min) | No movement | Barely moves | Moves but does not approach at least three sides of cage | Moves and approaches at least three sides of cage |
| symmetry of limb movement | Left side: no movement | Left side: slight movement | Left side: moves slowly | Both sides: move symmetrically |
| forepaw outstretching | Left side: no movement, no outreaching | Left side: slight movement to outreach | Left side: moves and outreaches less than right | Symmetrically outreach |
| climbing cage | ... | Fails to climb | Left side is weak | Normal climbing |
| body proprioception | ... | No response on left side | Weak response on left side | Symmetrical response |
| vibrissae reaction | ... | No response on left side | Weak response on left side | Symmetrical response |

**Supplementary Table S2.** The standard of neurological score of mice.
