# Supplementary figures and images for: Res@LDH: A Novel Nanohybrid Therapeutic for Ischemia–Reperfusion Injury with Dual Reactive Oxygen Species Scavenging Efficiency
Source: Biomater Res. 2024 Dec 3;28:0108. doi: 10.34133/bmr.0108 (PMC11612122; doi:10.34133/bmr.0108)

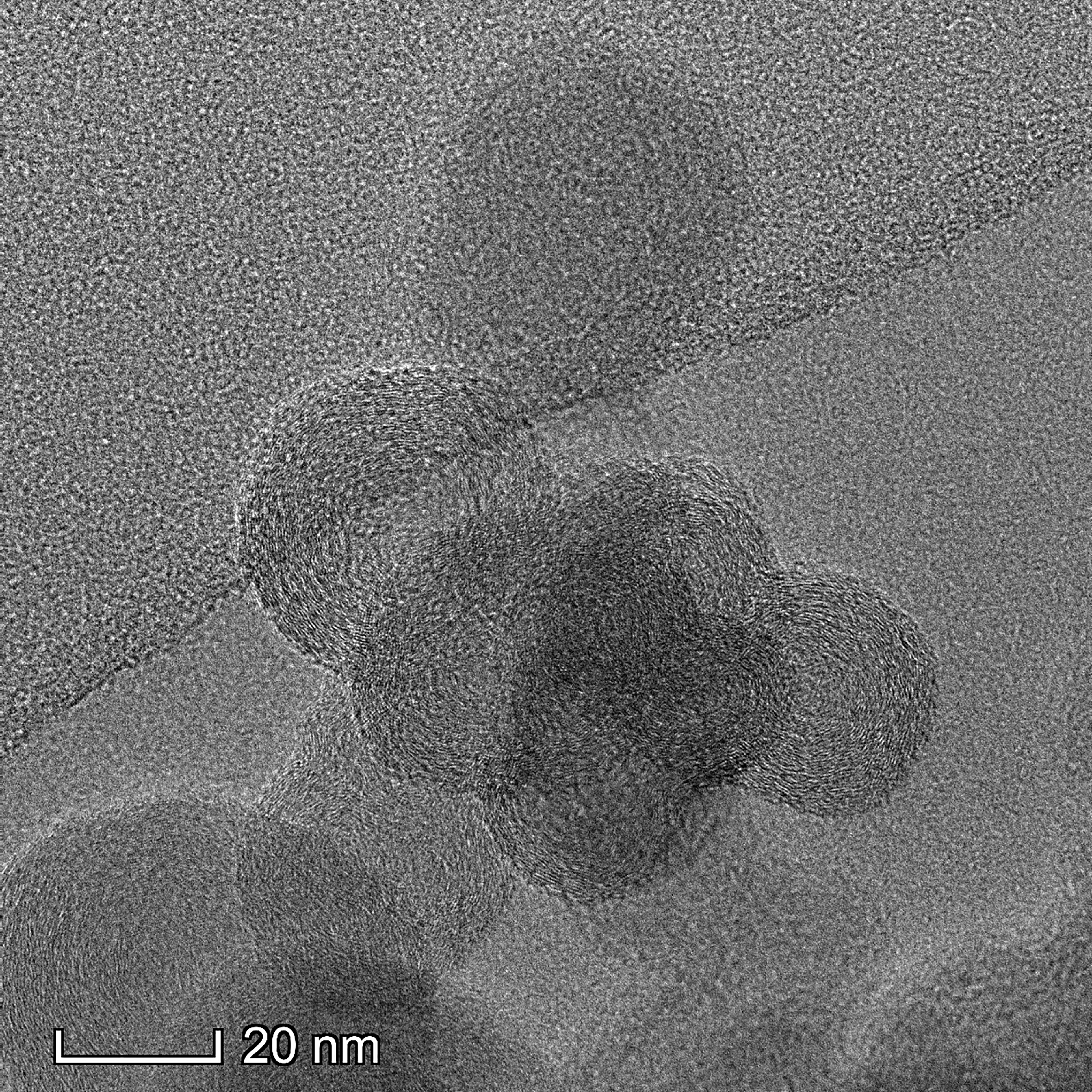

Supplement: Supplementary 1 — Figs. S1 to S5 Tables S1 and S2 [file bmr.0108.f1.zip › Supplementary Figure S1.tif]

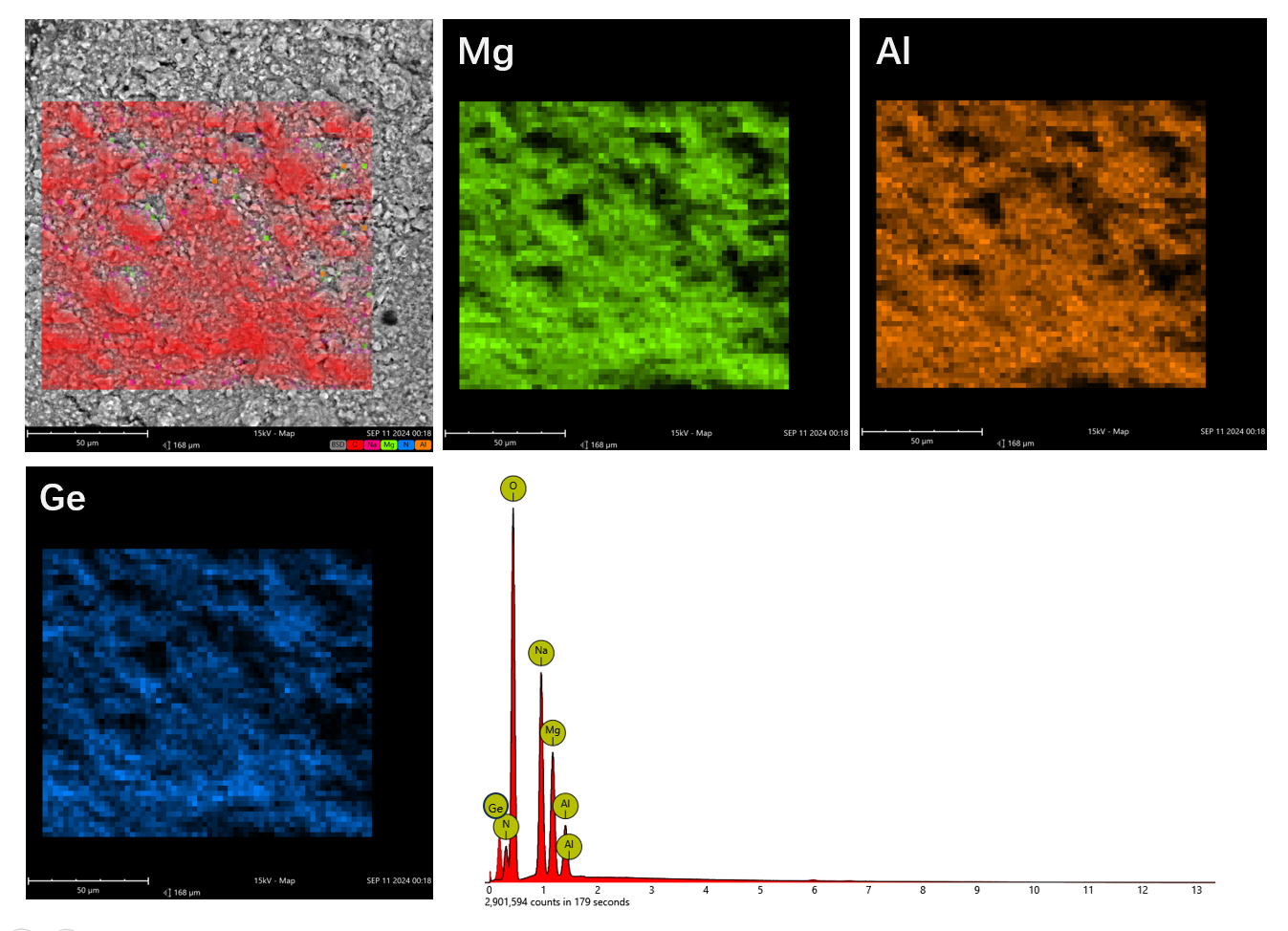

Supplement: Supplementary 1 — Figs. S1 to S5 Tables S1 and S2 [file bmr.0108.f1.zip › Supplementary Figure S2.tif]

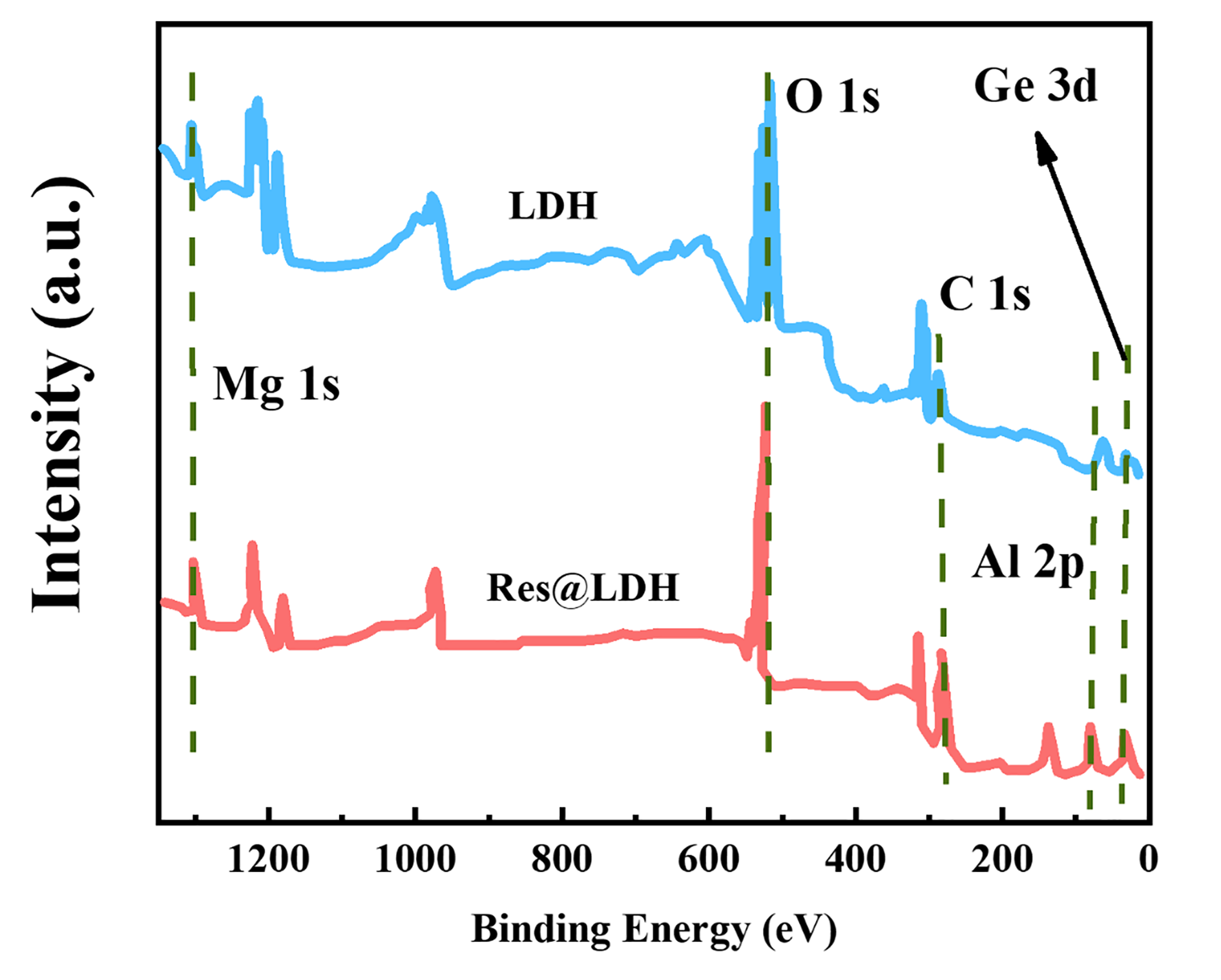

Supplement: Supplementary 1 — Figs. S1 to S5 Tables S1 and S2 [file bmr.0108.f1.zip › Supplementary Figure S3.tif]

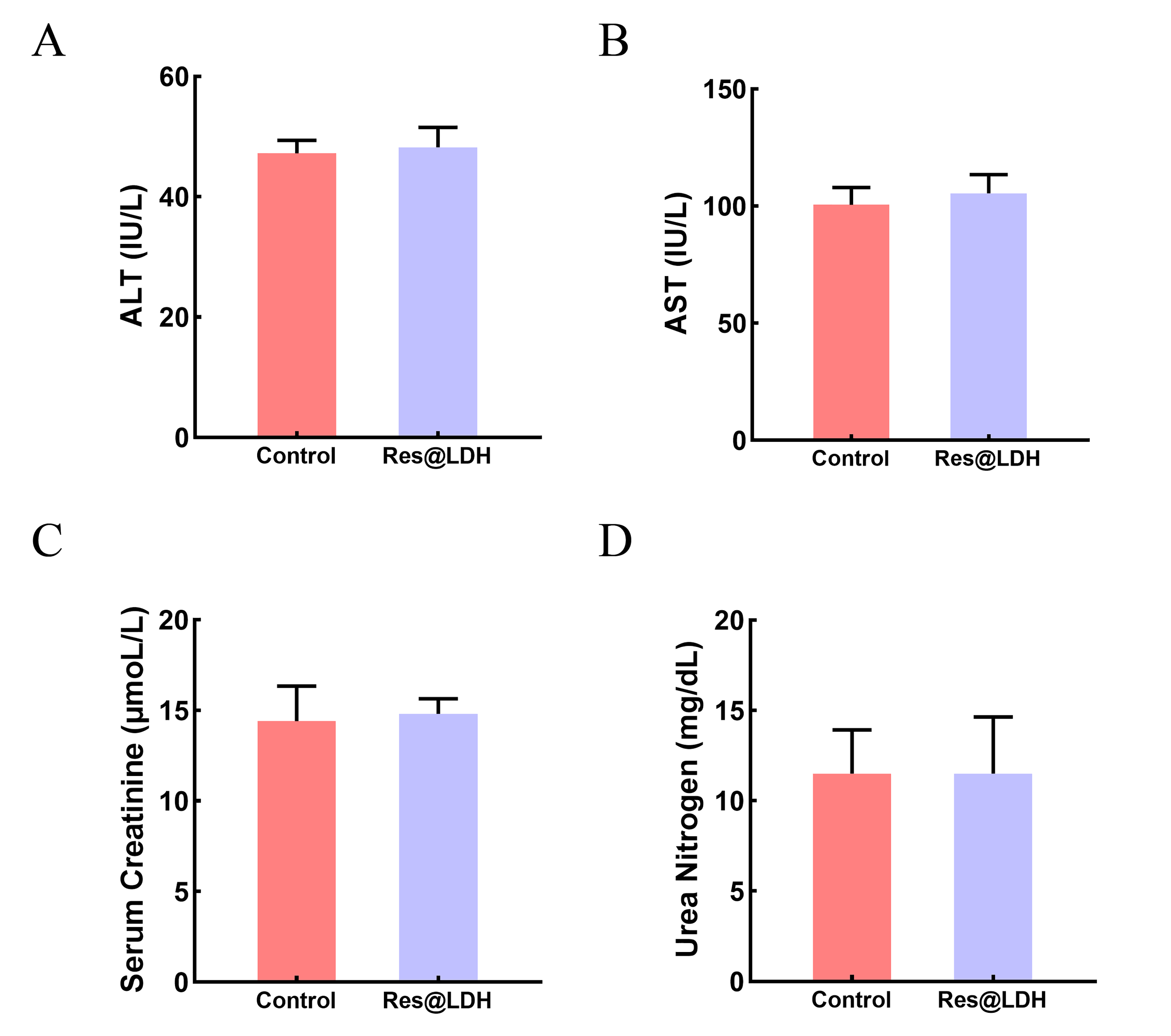

Supplement: Supplementary 1 — Figs. S1 to S5 Tables S1 and S2 [file bmr.0108.f1.zip › Supplementary Figure S4.tif]

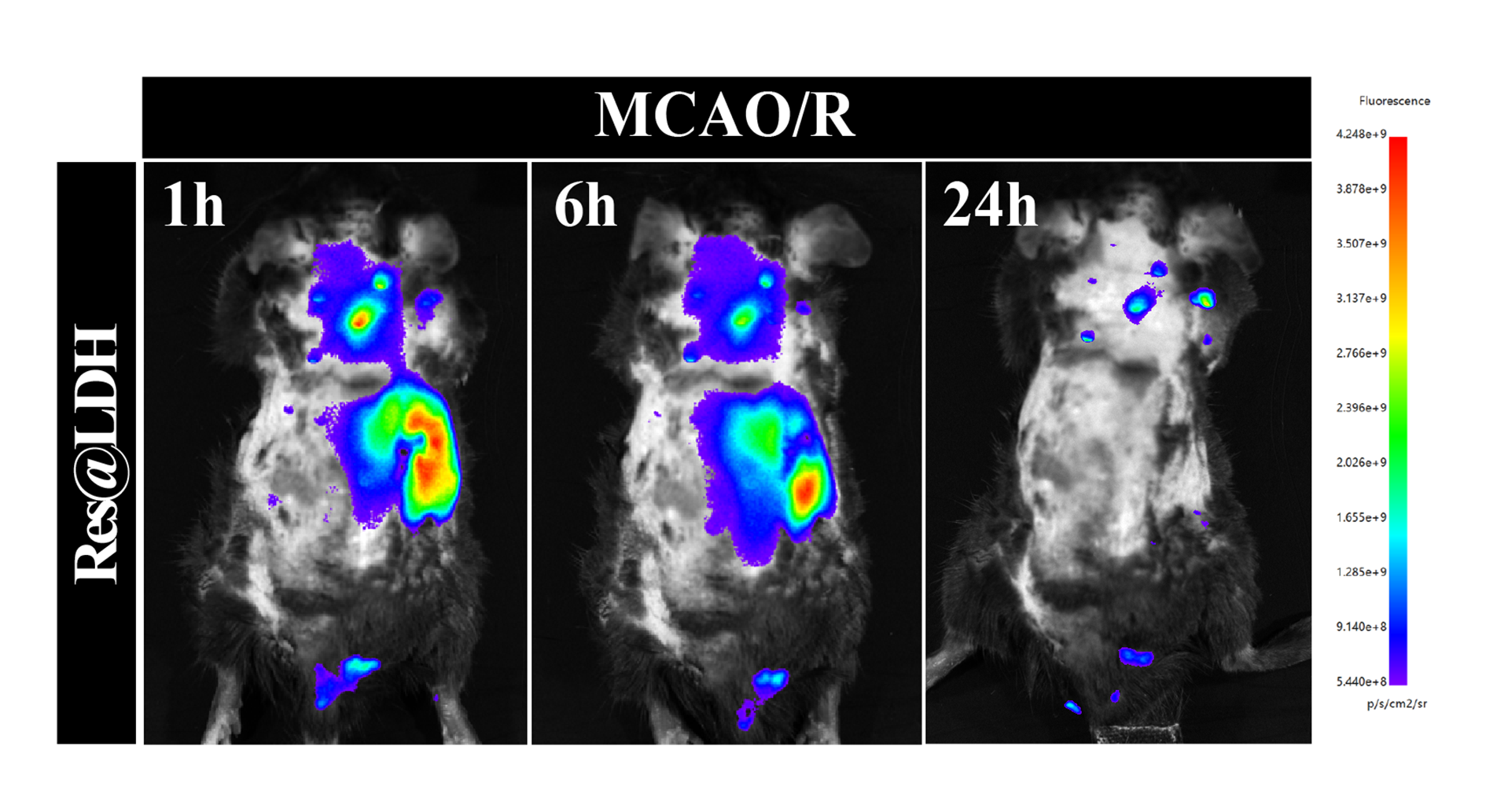

Supplement: Supplementary 1 — Figs. S1 to S5 Tables S1 and S2 [file bmr.0108.f1.zip › Supplementary Figure S5.tif]
